# Procedural efficiency vs clinical outcomes in persistent atrial fibrillation ablation: A population-level trade-off

**DOI:** 10.1016/j.hroo.2026.04.029

**Published:** 2026-06-04

**Authors:** Laurent Fauchier, Arnaud Bisson, Thibault Lenormand

**Affiliations:** Service de Cardiologie, Centre Hospitalier Universitaire Trousseau et Faculté de Médecine, Université François Rabelais, Tours, France

**Keywords:** Atrial fibrillation, Catheter ablation, Pulmonary vein isolation, Atrial tachycardia, Health care utilization, Population modeling

**Key Findings**

▪ In a capacity-constrained model (1000 electrophysiology laboratory h/y), a pulmonary vein isolation (PVI)–only strategy enabled treatment of more patients and resulted in fewer population-level cardiovascular hospitalizations than PVI with adjunctive ablation (PVI+) strategies.
▪ Although adjunctive ablation modestly reduces atrial fibrillation (AF) recurrence per patient, this benefit is offset by reduced procedural throughput and increased iatrogenic atrial tachycardia/flutter (“AT tax”).
▪ Across all modeled scenarios, PVI+ strategies were consistently associated with higher total atrial arrhythmia burden and cardiovascular hospitalizations, with no break-even point reached within realistic procedure durations.
▪ Patients not undergoing ablation owing to reduced capacity and treated with antiarrhythmic drugs contributed disproportionately to the overall arrhythmia burden and clinical events.
▪ These findings highlight the importance of evaluating AF ablation strategies beyond per-patient efficacy, incorporating system-level constraints and health care capacity.

Pulmonary vein isolation (PVI) is the cornerstone of catheter ablation for persistent atrial fibrillation (AF), yet recurrence remains frequent.^1^ PVI with adjunctive ablation (“PVI+”) strategies have been explored to improve outcomes, with mixed results across randomized trials.^1^, ^2^, ^3^ Although some approaches suggest reductions in AF recurrence, extensive ablation may increase procedural duration and promote iatrogenic atrial tachycardia (AT) or flutter (AFl).^1^ In an era of limited electrophysiology laboratory capacity and increasingly efficient pulsed field ablation (PFA), a population-level approach integrating procedural time, total atrial arrhythmia burden, and throughput seems warranted.

We developed a capacity-constrained model assuming a fixed availability of 1000 electrophysiology laboratory hours per year (Table 1). Procedural assumptions were based on contemporary PFA workflows, and clinical assumptions were derived from recent randomized trials and meta-analyses in patients with persistent AF with outcomes primarily assessed at 12 months. The mean duration of a PVI-only procedure was set at 60 minutes, whereas PVI+ strategies were assumed to increase procedural time by at least 15 minutes.^2^, ^3^, ^4^ This translated into an annual throughput of approximately 1000 patients for PVI and ∼320–800 patients for PVI+ (−20% to −65%, depending on procedural duration). Although adjunctive ablation may reduce AF recurrence, this benefit may be offset by incident AT/AFl. Accordingly, we modeled global atrial arrhythmia recurrence across varying scenarios (Table 1).

**Table 1:** Population-level cardiovascular hospitalizations according to ablation strategy (PVI only vs PVI+) in patients with persistent AF

Assumptions and results	PVI only	15-min PVI+	30-min PVI+	45-min PVI+	TAILORED-AF–style PVI+	PROMPT-AF–style PVI+
Assumptions						
Procedural duration (min)	60	75	90	105	170	188
EP laboratory time (h/y)	1000	1000	1000	1000	1000	1000
Patients ablated/y	1000	800	667	571	353	319
Patients on AAD	0	200	333	429	647	681
						
AF recurrence rate (12 mo)	∼25–35	∼24–35	∼24–35	∼24–35	14	24
AF/AT/AFl recurrence rate (12 mo)	∼30–45	∼27–44	∼27–44	∼27–44	40	27
AF recurrence rate if treated with AAD only	-	∼45–60	∼45–60	∼45–60	∼45–60	∼45–60
CV hospitalization rate after AF recurrence	∼70–80	∼70–80	∼70–80	∼70–80	∼70–80	∼70–80
CV hospitalization rate after ablation	∼21–36	∼19–35	∼19–35	∼19–35	∼28–32	∼19–21
CV hospitalization rate on AAD	-	∼32–48	∼32–48	∼32–48	∼32–48	∼32–48
						
Results						
AF/AT/AFl recurrence (ablated patients)	∼300–450	∼216–352	∼180–293	∼154–251	∼141–141	∼86–86
AF/AT/AFl recurrence (AAD patients)	-	∼90–120	∼150–200	∼193–257	∼291–388	∼306–409
Total AF/AT/AFl recurrence, estimated n (minimum–maximum)	∼375 (300–450)	∼389 (306–472)	∼412 (330–493)	∼428 (347–509)	∼481 (432–529)	∼443 (392–494)
Difference vs PVI only, n (%)	Reference	+ 14 (3.7)	+ 37 (9.8)	+ 53 (14.1)	+ 106 (28.2)	+ 68 (18.1)
						
CV hospitalizations (ablated patients)	∼210–360	∼151–282	∼126–235	∼108–201	∼99–113	∼60–68
CV hospitalizations (AAD patients)	-	∼63–96	∼105–160	∼135–206	∼204–311	∼214–327
Total CV hospitalizations, estimated n (minimum–maximum)	∼285 (210–360)	∼296 (214–378)	∼313 (231–395)	∼325 (243–407)	∼363 (303–424)	∼335 (274–395)
Difference vs PVI only, n (%)	Reference	+ 11 (3.8)	+ 28 (9.8)	+ 40 (14)	+ 78 (27.4)	+ 50 (17.5)

AAD = antiarrhythmic drug; AF = atrial fibrillation; AFl = atrial flutter; AT = atrial tachycardia; CV = cardiovascular; EP = electrophysiology; PVI = pulmonary vein isolation; PVI+ = pulmonary vein isolation with adjunctive ablation.

Cardiovascular hospitalization was estimated based on CAPLA post hoc analyses of cardiovascular health care utilization for recurrent atrial arrhythmias. Given that recurrence in persistent AF is often sustained and poorly tolerated, we assumed that 70%–80% of AF/AT recurrences result in cardiovascular hospitalization, translating arrhythmia burden into population-level outcomes. Patients not treated owing to reduced procedural capacity were assumed to receive antiarrhythmic drug therapy, with a recurrence rate of ∼45%–60% (Table 1).

This study was based on aggregated data from previously published studies and did not involve direct patient participation. Therefore, institutional review board approval and informed consent were not required.

Under a fixed annual electrophysiology capacity (1000 procedural hours), a PVI-only strategy using PFA (60 minutes per case) enabled treatment of 1000 patients, whereas PVI+ strategies reduced throughput depending on additional procedural time. With the estimated global atrial arrhythmia recurrence rates (AF + AT) leading to cardiovascular events, the PVI-only approach resulted in 375 AF/AT recurrences and 285 cardiovascular hospitalizations per year, serving as the reference strategy (Table 1).

When procedural duration with PVI+ increased to 75 minutes (+15 minutes), treatment capacity decreased to 800 patients, resulting in 389 AF/AT recurrences and 296 total cardiovascular hospitalizations, corresponding to a +3.8% hospitalization increase compared with PVI only (Table 1). With further prolongation to 90 minutes (+30 minutes), only 667 patients could be treated, leading to +9.8% hospitalizations vs PVI only (Table 1). With longer procedures, the reduction in throughput becomes even more pronounced, further widening the gap in favor of PVI only, given that patients not undergoing ablation and treated with antiarrhythmic drugs carry a higher recurrence burden. Across all scenarios, PVI+ strategies were consistently associated with higher AF/AT/AFl recurrences and population-level cardiovascular hospitalizations than PVI only. The excess for cardiovascular hospitalizations ranged from +3.8% to +14% and up to +27.4% with more recent and promising approaches and strategies. Overall, the break-even point was not reached within realistic procedural time increments (+15 to +30 minutes).

Waiting times for AF ablation represent a direct manifestation of system-level capacity constraints, with potential downstream effects on clinical outcomes.^5^ Although PVI+ may provide a modest per-patient reduction in AF recurrence, this benefit seems offset at the population level by reduced procedural capacity and the possible emergence of iatrogenic AT/AFl when linear lesions are incomplete or nondurable. Across all modeled scenarios, no PVI+ strategy achieved parity with PVI only in terms of total cardiovascular hospitalizations. These findings highlight the need to evaluate ablation strategies beyond per-patient efficacy, incorporating system-level constraints.

When total atrial arrhythmia burden—including AT/AFl—is accounted for, PVI only consistently results in fewer cardiovascular hospitalizations at the population level, establishing it as the more efficient strategy under constrained resources. When integrated into a capacity-constrained framework, this “AT tax” reverses the apparent advantage of PVI+, making PVI only the more efficient strategy at the population level in persistent AF. The key driver is the combination of minimal net efficacy gain (∼0.5%) and substantial throughput reduction (20%–25%).

Importantly, this conclusion may not be universal. An exception may arise with optimized substrate modification using a PROMPT-AF–like strategy, where durable mitral isthmus block reduces AT burden and yields clinically meaningful benefit, provided that procedural duration remains limited. In such settings, the per-patient gain may exceed the threshold required to offset reduced procedural capacity.

Among limitations, this analysis relies on simplified assumptions, limited durability data for PFA lesions, and short-term outcomes, with potential variability in real-world practice and uncertainty in translating recurrence to clinical events.

Overall, although relying on simplified assumptions, these findings support selective rather than systematic use of PVI+ for catheter ablation of persistent AF at the population level. Future studies should incorporate total atrial arrhythmia burden, procedural efficiency, and global health care utilization to better guide ablation strategy in modern electrophysiology practice.

## Disclosures

L.F.: consultant or speaker fees of small amounts for AstraZeneca, Bayer, Bristol Myers Squibb/Pfizer, Boehringer Ingelheim, Boston Scientific, Medtronic, Novo Nordisk, Servier, and Zoll outside of this work. A.B.: consultant or speaker for AstraZeneca, Bayer, Bristol Myers Squibb/Pfizer, Medtronic, Vifor Pharma, and Alnylam. The other author has no conflicts of interest to disclose.
